# Social determinants of health and COVID-19: An evaluation of racial and ethnic disparities in attitudes, practices, and mental health

**DOI:** 10.1371/journal.pgph.0000558

**Published:** 2023-01-23

**Authors:** Jenil R. Patel, Clare C. Brown, T. Elaine Prewitt, Zain Alfanek, M. Kathryn Stewart

**Affiliations:** 1 Department of Epidemiology, Human Genetics and Environmental Sciences, The University of Texas Health Science Center at Houston (UTHealth) School of Public Health, Dallas, Texas, United States of America; 2 Department of Epidemiology, Fay W. Boozman College of Public Health, University of Arkansas for Medical Sciences, Little Rock, Arkansas, United States of America; 3 Department of Health Policy and Management, Fay W. Boozman College of Public Health, University of Arkansas for Medical Sciences, Little Rock, Arkansas, United States of America; 4 Department of Internal Medicine, UAMS College of Medicine, University of Arkansas for Medical Sciences, Little Rock, Arkansas, United States of America; University of Colorado Denver - Anschutz Medical Campus: University of Colorado - Anschutz Medical Campus, UNITED STATES

## Abstract

Previous evaluations have reported racial minorities feel they are at greater risk of contracting COVID-19, but that on average, they have better preventative practices, such as wearing face masks and avoiding large gatherings. In this study, we explored associations between social determinants of health (SDOH), race and ethnicity, COVID-19 practices and attitudes, and mental health outcomes during the pandemic. We examined associations between SDOHs and practices, attitudes, and mental health symptoms by race and ethnicity using multivariable linear and logistic regressions in 8582 Arkansan pulse poll respondents (September—December, 2020). Compared to White respondents, mean attitude and practice scores were greater (indicating safer) among Black (4.90 vs. 3.45 for attitudes; 2.63 vs. 2.41 for practices) and Hispanic respondents (4.26 vs. 3.45 for attitudes; 2.50 vs. 2.41 for practices). Respondents’ SDOH scores by race/ethnicity were: White (3.65), Black (3.33), and Hispanic (3.22). Overall, attitude and practice scores decreased by 0.35 and 0.09, respectively, for every one-point increase in SDOH. Overall, a one-point increase in SDOH was associated with 76% and 85% increased odds of screening negative for anxiety and depression, respectively. To conclude, underlying social inequities are likely driving safer attitudes, practices, and worse anxiety and depression symptoms in Black and Hispanic Arkansans. In terms of policy implications, our study supports the urgency of addressing SDOHs for rural states similar to Arkansas.

## Introduction

The U.S. leads all other nations in the number of coronavirus disease 19 (COVID-19) cases, with over 30 million cases and over 550,000 deaths as of April 8, 2021 [1]. The pandemic has brought racial and ethnic inequities to the forefront, with Black/African American (“Black”, hereafter) and Hispanic/Latinx (“Hispanic” hereafter) populations in the U.S. both having 3 times the hospitalizations and 2 times the rates of death from COVID-19, compared to White individuals [2]. The cause of racial and ethnic disparities is multifactorial, and include lower rates of insurance coverage [3], and less consistent management of comorbidities such as diabetes and hypertension which increase the risk of death from the virus [4]. Evidence also shows that chronic stress due to discrimination and implicit biases in the clinical setting contribute to the problem of reduced access to care and lower overall health prior to the pandemic [5, 6]. The disproportionate representation of Black and Hispanic individuals in occupations posing higher risk of COVID-19 exposure results from structural racism, which powerfully binds race and ethnicity to socioeconomic status and occupation [7, 8].

Previous evaluations have reported racial minorities feel at greater risk of contracting COVID-19, but on average, they have better preventative practices, such as wearing face masks and avoiding large gatherings [9–11]. This study builds on this existing literature by examining how race and ethnicity, together with social determinants of health (SDOH), affect respondents’ attitudes and practices about protective policies related to COVID-19. Specifically, our evaluation utilizes data from a random digit pulse poll created by the University of Arkansas for Medical Sciences (UAMS) Fay W. Boozman College of Public Health (COPH) to design a SDOH score and to assess whether SDOHs impact COVID-19 practices and attitudes among individuals of different races and ethnicities.

Arkansas consistently ranks among states with poorest health outcomes, and Black and Hispanic populations experience higher rates of morbidity and mortality from several comorbid conditions, including cancer [12], diabetes [13], and heart disease [14]. This unfavorable situation ultimately creates a context for evaluating how SDOHs may differentially impact adults of different races and ethnicities. In Arkansas, for example, the UALR public radio report by Arkansas Advocates for Children and Families showed worse health outcomes for a number of conditions are more likely in Black women. Intersectionality experienced by Black women have in the United States is a primary reason these populations are disproportionately affected.

In the context of the COVID-19 pandemic, Arkansas has ranked higher among states with the highest COVID-19 transmission starting in July of 2020 according to the White House Coronavirus Task Force [15].

## Methods

### Study data and population

We used data collected from the Pandemic Pulse Poll, initiated by the UAMS COPH from May to December 2020 using a random digit dialing of ground telephones and cell phone numbers in Arkansas (n = 13,057). The phone numbers were purchased from a national company with telephone polling experience in Arkansas. Cellphone numbers were targeted based on usage, as determined by call volume and locations most used by a cellphone. Cell numbers not currently in use or with high out-of-state usage during study period were excluded from the list of numbers provided. Trained research assistants (RA) contacted participants and asked standardized questions about COVID-19.

In order to participate, respondents were required to be a resident of Arkansas, age 18+, and able to provide consent. If respondents were Spanish-speaking, the interview was conducted in Spanish. All responses were recorded using REDCap survey software.

Data for the current study are a subset of the entire 13,057 respondent sample. Specifically, this analysis is limited to data from September through December (n = 9,470) due to changes in key study variables. Participants were excluded from our analyses who did not identify as White, Black, or Hispanic (n = 196 excluded) resulting in a final sample of 9,274. Among those, 692 individuals were removed for missing information, resulting in a final study sample of 8,582.

All participants provided a verbal consent prior to the administration of survey. The study was approved and deemed non-human subjects research by the UAMS IRB and was registered on their official IRB database (UAMS IRB#262928).

### Variable definitions

Demographic characteristics included sex (male/female), age (<60y, > = 60y), self-reported race/ethnicity (White, Black, and Hispanic), and rurality (rural [levels 4–9 Rural-Urban Commuting Area Codes], urban) [16].

#### Pandemic attitudes and practices

Participants answered questions about attitudes and preventative practices in regard to the pandemic. An *attitude score* was created by summing eight binary-transformed variables, such that eight represents the most safe attitudes. These variables included wanting to be tested for COVID-19 (yes/no/don’t know), feeling that testing is important (from not important to most important on scale 1–5), recognizing that shopping and eating in restaurants may not be safe (from ‘Very unsafe’ to ‘Very safe’ on scale 1–5), non-optimism regarding the pandemic ending soon (from ‘Not optimistic at all’ to ‘Very optimistic’ on scale 1–5), disagreeing with opening public schools (from ‘Strongly disagree’ to ‘Strongly agree’ on scale 1–5), disagreeing with allowing large gatherings (from ‘Strongly disagree’ to ‘Strongly agree’ on scale 1–5), agreeing that masks stop the spread of COVID-19 (yes/no/don’t know), and agreement that the state needs a mask order (yes/no). A *practices score* was created by summing three variables related to higher risk practices, such that a value of three represents those with the least risky practices. These variables included regularly wearing masks (yes/no), not attending a religious gathering in the last two weeks (yes/no/don’t know), and not attending a family/community event with 10+ people in the last two weeks (yes/no/don’t know).

#### Social determinants of health

A 4-item *social-determinants of health (SDOH) score* was created by summing 4 binary-coded variables representing beneficial social determinants, such that a score of 4 represents an individual with the best circumstances for SDOHs. The SDOH score included the response about (i) not feeling worried about the ability to see a doctor if sick (from ‘Very worried’ to ‘Not worried at all’ on a scale of 1–5), (ii) having enough money for food in the last two weeks (yes/no/don’t know), (iii) not being worried about paying for food in the last two weeks (from ‘Very worried’ to ‘Not worried at all’ on a scale of 1–5), and (iv) not being worried about having enough money to pay the rent or mortgage in the last two weeks (from ‘Very worried’ to ‘Not worried at all’ on a scale of 1–5).

#### Mental health outcomes during the pandemic

We constructed two measures of mental health, including a measure of depression symptoms and a measure of anxiety symptoms over the last 7 days (rather than the usual 14 days) [17]. The depression symptoms measure included the Patient Health Questionnaire-2 (PHQ). The two questions asked about having little interest or pleasure in doing things and about feeling down, depressed, or hopeless. The anxiety measure included two questions that form the two-item Generalized Anxiety Disorder-2 (GAD) score, which asks respondents to report whether in the last 7 days they felt nervous, anxious, or on edge and whether the respondent felt unable to control worrying.

We ensured the directions of opposite responses (for e.g. feeling that testing is important versus recognizing that shopping and eating in restaurants may not be safe) were accounted for while developing the scores.

### Statistical analysis

Descriptive statistics included chi-square tests for categorical covariates and Wald tests for continuous variables. First, multivariable linear regression was conducted to assess the association of sex, age, rurality, and race with SDOH scores. Separate regression analyses were conducted for each race, while controlling for sex, age, and rurality. Second, regression models were used to assess the association of SDOH scores with COVID-19 attitudes and practices as well as with the two measures of mental health (anxiety symptoms and depression symptoms), adjusting for age, sex, race, and rurality. We subsequently assessed differences in the association of SDOH and each of the outcomes (attitudes, practices, and mental health outcomes) using regressions stratified by race and ethnicity. Linear regressions were used for models with attitudes and practices as outcomes, and logistic regressions were used for models with anxiety or depression symptoms as an outcome. All analyses were conducted using STATA v15 (StataCorp, College Station, TX). Analyses were weighted based on age, race, and sex to be representative of the Arkansas population.

## Results

Table 1 provides demographic characteristics of the study sample. Of the 8,582 participants, 76.4% (n = 6,552) were Whites, 18.2% (n = 1,560) were Blacks and 5.5% (n = 470) were Hispanics. Table 1 also provides the percent distributions of sex, age, and rurality, and mean attitudes, practices, and social determinants of health (SDOH) scores overall and among each race/ethnicity. Compared to Whites, the mean attitude and practice scores were greater among Blacks (4.90 vs. 3.45 for attitudes; 2.63 vs. 2.41 for practices) and among Hispanics (4.26 vs. 3.45 for attitudes; 2.50 vs. 2.41 for practices). SDOH scores were the greatest among Whites (3.65), followed by Blacks (3.33) and Hispanics (3.22). Among all races, the proportion of respondents screening negative for generalized anxiety disorder ranged from 82.5% to 83.2%, and the percent of respondents negative for depressive symptoms ranged from 85.7% to 87.5%. The differences for these proportions among races were not statistically significant.

**Table 1:** Demographic characteristics of individuals participating in random digit dial pulse poll in Arkansas from September 5, 2020 to December 18, 2020, overall and by race and ethnicity^a^.

Characteristic		Statistic	Overall (N = 8582)	Race / Ethnicity			
				White (N = 6552)	Black (N = 1560)	Hispanic (N = 470)	p-value*^a^
Sex							
	Female	%	51.6	50.9	56.2	44.9	<0.01
	Male		48.4	49.1	43.8	55.1	
Age							
	<60	%	70.2	66.7	76.7	97.3	<0.01
	≥60		29.8	33.3	23.4	2.7	
Rurality							
	Rural	%	55.9	57.3	52.6	45.9	<0.01
	Urban		44.1	42.7	47.4	54.2	
Attitudes (0 to 8)		Mean (SE)	3.75 (0.03)	3.45 (0.03)	4.90 (0.06)	4.26 (0.13)	<0.01
Practices (0 to 3)		Mean (SE)	2.46 (0.01)	2.41 (0.01)	2.63 (0.02)	2.50 (0.04)	<0.01
SDOH (0 to 4)		Mean (SE)	3.57 (0.01)	3.65 (0.01)	3.33 (0.03)	3.22 (0.06)	<0.01
Generalized Anxiety Disorder		%					0.95
	Yes		17.1	17.2	16.8	17.5	
	No		82.9	82.9	83.2	82.5	
Symptoms of Major Depression		%					0.20
	Yes		12.9	12.5	14.3	14.2	
	No		87.1	87.5	85.7	85.8	

^a^ Analyses weighted based on distribution of age, race, and sex among Arkansas residents.

*p-value corresponds to tests for differences in values among respondents of different race/ethnicities using

Chi-square test for sex, age, rurality, generalized anxiety disorder, and major depressive disorder, and Wald test for attitudes, practices, and SDOH scores.

Table 2 provides the adjusted associations of demographic factors and SDOH scores overall and stratified by race/ethnicity. The intercept refers to the value when all independent covariates are equal to the reference value. The intercepts for mean SDOH scores were 3.33, 3.35, 2.95, and 2.87, for overall, White, Black, and Hispanic respondents, respectively. SDOH scores were higher (better social determinants) for older respondents (0.13, 95% confidence interval (CI) 0.10,0.16) overall, as well as among Whites (0.12, 95%CI 0.09,0.16) and Blacks (0.15, 95%CI 0.05,0.26) respondents. SDOH scores were higher in rural populations among Whites (0.04, 95%CI 0.01,0.18) but not among Black and Hispanic respondents. Among the regression with all respondents, SDOH scores were lower for Black (-0.30, 95%CI -0.36,-0.25) and for Hispanic (-0.40, 95%CI -0.51,-0.28) respondents, compared to White respondents.

**Table 2:** Adjusted association of demographic factors with SDOH scores among Arkansans overall and by race and ethnicity from September 5, 2020 to December 18, 2020^a^^,^^b^.

		Overall		White		Black		Hispanic	
		Coef (95% CI)	p-value	Coef (95% CI)	p-value	Coef (95% CI)	p-value	Coef (95% CI)	p-value
Sex									
	Female	Ref	Ref	Ref	Ref	Ref	Ref	Ref	Ref
	Male	0.09 (0.06, 0.13)	<0.01	0.08 (0.05, 0.12)	<0.01	0.14 (0.03, 0.25)	0.01	0.02 (-0.20, 0.24)	0.84
Age									
	<60	Ref	Ref	Ref	Ref	Ref	Ref	Ref	Ref
	>60	0.13 (0.10, 0.16)	<0.01	0.12 (0.09, 0.16)	<0.01	0.15 (0.05, 0.26)	0.01	0.36 (-0.14, 0.85)	0.16
Rurality									
	Rural	Ref	Ref	Ref	Ref	Ref	Ref	Ref	Ref
	Urban	0.02 (-0.01, 0.06)	0.23	0.04 (0.01, 0.08)	0.02	-0.03 (-0.14, 0.08)	0.59	-0.09 (-0.32, 0.14)	0.45
Race									
	White	Ref	Ref	-	-	-	-	-	-
	Black	-0.30 (-0.36, - 0.25)	<0.01	-	-	-	-	-	-
	Hispanic	-0.40 (-0.51, -0.28)	<0.01	-	-	-	-	-	-

^a^ Linear regression analyses weighted based on distribution of age, race, and sex among Arkansas residents.

^b^ Intercepts: Overall model = 3.33, White = 3.35, Black = 2.95, Hispanic = 2.87

Table 3 shows adjusted associations of SDOH scores with attitudes, practices, and mental health outcomes during the pandemic. The mean intercepts for attitude scores and practice scores for overall models adjusted for age, sex, rurality and race, were 4.37 and 2.61 respectively. Overall, a unit increase in the SDOH score resulted in a decrease in attitude and practices scores by 0.35 and 0.09, respectively. A one unit increase in SDOH score was associated with significant declines (less safe) in attitude scores among all White (-0.39, 95%CI -0.49,-0.29), Black (-0.28, 95%CI -0.41,-0.16), and Hispanic (-0.27; 95%CI -0.53,-0.01) populations. Decreases in practices scores (less safe) were significant among White (-0.12, 95%CI -0.14,-0.10) and Hispanic (-0.08; 95%CI -0.15,-0.00) populations, but not among Blacks (-0.02; 95%CI -0.05,0.01). We found significant associations of SDOH with mental health outcomes overall, and among White and Black respondents, but not for Hispanic respondents. Overall, a one unit increase in the SDOH score was associated with a 76% increase in the odds of being screened negative for symptoms of anxiety and 66% increase in the odds of being screened negative for depressive symptoms. SDOH scores were associated with increased odds of screening negative for anxiety and depression among White (aOR:1.91; 95%CI: 1.75,2.09 and aOR: 1.99, 95%CI: 1.82,2.18) and among Black respondents (aOR:1.67; 95%CI: 1.46,1.92 and aOR: 1.76, 95%CI: 1.53,2.02).

**Table 3:** Association of SDOH scores with attitudes, practices and mental health outcomes among Arkansans overall and by race and ethnicity ^a^^,^^b^^,^^c^^,^^d^.

Type of Score	Coefficient Type	Overall		White		Black		Hispanic	
		Measure of Association (95% CI)	p-value	Measure of Association (95% CI)	p-value	Measure of Association (95% CI)	p-value	Measure of Association (95% CI)	p-value
Attitudes	β	-0.35 (-0.42, -0.27)	<0.01	-0.39 (-0.49, -0.29)	<0.01	-0.28 (-0.41, -0.16)	<0.01	-0.27 (-0.53, -0.01)	0.04
Practices	β	-0.09 (-0.11, -0.07)	<0.01	-0.12 (-0.14, -0.10)	<0.01	-0.02 (-0.05, -0.01)	0.26	-0.08 (-0.15, -0.00)	0.04
Screened Negative for Generalized Anxiety Disorder	Odds Ratio	1.76 (1.64–1.89)	<0.01	1.91 (1.75–2.09)	<0.01	1.67 (1.46–1.92)	<0.01	1.12 (0.86–1.46)	0.39
Screened Negative for Depression	Odds Ratio	1.85 (1.72–2.00)	<0.01	1.99 (1.82–2.18)	<0.01	1.76 (1.53–2.02)	<0.01	1.30 (0.97–1.75)	0.08

^a^ Attitudes and practices were evaluated using linear regressions adjusted for race (“overall” analysis only), age, sex, and rurality.

^b^ Attitude scores Intercepts: Overall = 4.37, White = 4.61, Black = 5.34, Hispanic = 3.96

^c^ Practice score Intercepts: Overall = 2.61, White = 2.73, Black = 2.68, Hispanic = 2.22

^d^ Generalized anxiety disorder and symptoms of depression were evaluated using logistic regressions adjusted for race (“overall” analysis only), age, sex, and rurality. Analyses were weighted based on distribution of age, race, and sex among Arkansas residents.

## Discussion

This study used data from a random digit dial pulse poll related to COVID-19 and found significant racial and ethnic disparities in conditions such as access to care, and housing/food insecurity defining social determinants of health. Black and Hispanic respondents had significantly lower SDOH scores, meaning situations less conducive to good health, but had safer COVID-related attitudes and practices compared to White respondents. Additionally, respondents with better SDOH scores had lower odds of symptoms of anxiety and depression and more risky attitudes and practices related to COVID-19, regardless of race.

We found relatively lower SDOH scores among Black and Hispanic populations relative to White respondents, suggesting that such minority populations are more likely to be in situations that require safer practices. To affirm these findings, one example is in the state of Kansas that shares similar demographics with Arkansas, where by June 2020, according to the COVID Racial Data Tracker, only 4,854 from over 94,000 tests were from black Americans, while 50,070 were from whites [18]. However, blacks make up almost a third of the state’s COVID-19 deaths [18]. Assessments of the 95%CI in adjusted analyses do not indicate that social determinants of health had a stronger association with attitudes and practices or with mental health outcomes among any specific racial group. However, we do see an important relationship with respect to mental health outcomes worth noting. Specifically, the odds of screening negative for anxiety or depression, indicating better mental health outcomes, among White respondents falls outside of the 95%CI among Black respondents, and the opposite is true as well. While not causal in interpretation, this suggests that other facets of institutional racism and structural biases may be driving Black-White disparities in mental health outcomes. Specifically, Black respondents had worse mental health outcomes relative to White respondents, regardless of SDOH.

The unequal distribution of these adverse SDOH among racial and ethnic minority groups, including but not limited to African American and Hispanics, is a defining feature of structural racism [19]. These findings contribute to the ever-expanding body of research documenting racial and ethnic disparities in COVID-19 infection, hospitalization, and mortality rates by deepening our understanding of the role of the social determinants in these outcomes. Much of this literature identifies structural racism as the enduring, underpinning cause of higher rates among Black and Hispanic communities [20–23]. Structural racism creates inequities in opportunities to live in well-resourced neighborhoods [24, 25], to have opportunities for healthy affordable housing, quality education, safety, food security, transportation, and other SDOH [26]. Indeed, during this pandemic these inequities have also created unequal access to testing, early diagnosis, and sufficient treatment resources for Black and Hispanic individuals, who are less likely to have insurance coverage and more likely to face access to care issues related to Medicaid coverage [3, 26].

Previous studies have examined racial differences in perceived risk, knowledge, attitudes, and practices related to COVID-19. Niño et al., found that Black, Hispanic, and Asian American individuals were more likely to perceive coronavirus as a major threat to their health relative to White individuals; the authors posit that differences relate to structural disadvantages affecting Black, Hispanic, and Asian Americans at higher rates than Whites [9]. A study of attitudes and practices related to COVID-19 found more Blacks and Hispanics than Whites to have reported worries about financial issues and access to healthcare. They found that Black and Hispanic respondents had a lower level of knowledge regarding COVID-19, such as public health preventative recommendations, disease risk factors and symptomatology compared to Whites, but Black and Hispanic respondents had better practices, such as mask wearing and avoiding travel and large gatherings [10]. Our findings on safer attitudes and practices among Blacks and Hispanics are consistent with these findings. The complexity of these issues deserves further exploration given the increased stigma and discrimination experienced by racial and ethnic minorities during the pandemic; with some Black individuals reporting concerns that wearing a mask will increase their risk of racial profiling and harassment by law enforcement [27].

We found similar perceptions of being at high risk for contracting coronavirus among Whites (27%), Blacks (29%), and Hispanics (27%). While we did not find racial and ethnic differences, it is conceivable that the historically relentless and extensive structural barriers experienced by Blacks and Hispanics may have led them to be more risk-averse in terms of their attitudes and practices. On the other hand, Wolf et al., found Blacks were less worried and felt less likely to get sick with the coronavirus compared to White respondents [11]. A small percentage of Black respondents in their study reported changing their daily routine because of COVID-19; however, the difference was not significant when adjusted for other demographics, health literacy, and day of the survey [11].

Racial and ethnic disparities in adverse mental health related to COVID-19 have also been studied. Czeisler et al. documented poor mental health, increased substance use, and more suicidal ideation related to the pandemic among racial and ethnic minorities and essential workers in the U.S. [28]. In the national household pulse survey on COVID-19 conducted by the National Center for Health Statistics, higher percentages of Blacks and Hispanics than Whites reported symptoms of anxiety and depressive disorder during the last 7 days [18]. We found that improved SDOH was less likely to improve mental health outcomes among Hispanics compared to Whites and Blacks. This finding is perhaps not surprising given some sensitive factors and concerns we were not able to capture with our SDOH score such as fears of deportation and lack of legal protections from exploitation [29, 30].

Our study had several limitations. First, the study did not have a sufficient number of participants from other underrepresented races and ethnicities to include in our analyses. While the study was not designed to oversample smaller populations, future studies are needed to learn more about the pandemic among other high-risk populations in the state, particularly the Marshallese, for whom data on COVID-19 risk behaviors, attitudes and SODH factors is clearly needed. Additionally, the relatively small sample size likely limited our conclusions regarding differences in associations between SDOH and mental health outcomes among individuals of different races. Specifically, the standard errors in adjusted regressions were large and limited comparisons of stratified analyses. Second, due to changes in the study questions in September, we were not able to evaluate changes in attitudes, practices, and mental health over time. Moreover, due to the emerging nature of the pandemic, our study might not have captured all the SDOH variables that are specific to pandemic and the affected minority communities.

The results of our study should also be interpreted within the context of the timeframe of the study; specifically, our data collection ended before introduction of the new COVID-19 vaccine, prior to discovery of new variants of the coronavirus in the US, and before the inauguration of the Biden administration. Therefore, our results do not reflect how attitudes and practices related to masking [31] and avoiding gatherings may be changing in this context.

Several strengths of the study should be noted. The demographic characteristics of Arkansas provide insight about behaviors, attitudes, mental health and SDOH among the highest risk populations in the state, particularly rural populations who historically have limited access to prevention and treatments that mitigate impact of the virus. These findings are relevant for other states with significant racial and ethnic disparities, particularly those with large rural populations similar to Arkansas. Additionally, the data were weighted based on race, age, and sex using population-based estimates from Arkansas and therefore findings are representative of the state. Moreover, the survey was a random digit dial survey available to both English and Spanish speakers; calls were made during weekday hours, evenings, and weekends to capture working and non-working populations equally. Finally, the survey was adapted based on the Household Pulse Survey conducted by the National Center for Health Statistics (NCHS) and the Census Bureau. As a state-specific poll, we were able to change questions to query about specific policies and mandates underway in Arkansas to learn about their COVID-19 related attitudes, practices, and mental health.

## Public health implications

The COVID-19 crisis is exacting a devastating toll on population health. Our findings support the urgency of addressing SDOHs in rural states similar to Arkansas. Comprehensive, culturally appropriate strategies addressing public awareness, education, engagement, and access to services targeting high-risk and under-reached Black and Hispanic communities must be central components of effective state-level public health emergency preparedness policies to abate the pandemic.

Social determinants are driving crucial negative events, including differential exposure to COVID, susceptibility to infection, and overall consequences of infection [32]. Egede and Walker propose actions to address the impact of structural racism on health including policy change, cross-sector partnerships, economic empowerment, financial support for community programs, trust-building in communities affected, and interventions that target social risk factors [23]. Attention to such equity focused efforts is urgently needed to address these concerns.
